# Methylation Density Pattern of *KEAP1* Gene in Lung Cancer Cell Lines Detected by Quantitative Methylation Specific PCR and Pyrosequencing

**DOI:** 10.3390/ijms20112697

**Published:** 2019-05-31

**Authors:** Federico Pio Fabrizio, Angelo Sparaneo, Flavia Centra, Domenico Trombetta, Clelia Tiziana Storlazzi, Paolo Graziano, Evaristo Maiello, Vito Michele Fazio, Lucia Anna Muscarella

**Affiliations:** 1 Laboratory of Oncology, Fondazione IRCCS Casa Sollievo della Sofferenza, 71013 San Giovanni Rotondo, Italy; a.sparaneo@operapadrepio.it (A.S.); f.centra@operapadrepio.it (F.C.); d.trombetta@operapadrepio.it (D.T.); FAZIO@unicampus.it (V.M.F.); l.muscarella@operapadrepio.it (L.A.M.); 2 Department of Biology, University of Bari “Aldo Moro”, 70125 Bari, Italy; cleliatiziana.storlazzi@uniba.it; 3 Unit of Pathology, Fondazione IRCCS Casa Sollievo della Sofferenza, 71013 San Giovanni Rotondo, Italy; p.graziano@operapadrepio.it; 4 Department of Onco-Haematology, Fondazione IRCCS Casa Sollievo della Sofferenza, 71013 San Giovanni Rotondo, Italy; e.maiello@operapadrepio.it; 5 Department of Medicine, Laboratory of Molecular Medicine and Biotechnology, University Campus Bio-Medico of Rome, 00128 Rome, Italy

**Keywords:** *KEAP1*, methylation, lung cancer, QMSP, pyrosequencing

## Abstract

Background. The KEAP1/NRF2 pathway is the key regulator of antioxidants and cellular stress responses, and is implicated in neoplastic progression and resistance of tumors to treatment. *KEAP1* silencing by promoter methylation is widely reported in solid tumors as part of the complex regulation of the KEAP1/NRF2 axis, but its prognostic role remains to be addressed in lung cancer. Methods. We performed a detailed methylation density map of 13 CpGs located into the *KEAP1* promoter region by analyzing a set of 25 cell lines from different histologies of lung cancer. The methylation status was assessed using quantitative methylation specific PCR (QMSP) and pyrosequencing, and the performance of the two assays was compared. Results. Hypermethylation at the promoter region of the *KEAP1* was detected in one third of cell lines and its effect on the modulation *KEAP1* mRNA levels was also confirmed by in vitro 5-Azacytidine treatment on lung carcinoid, small lung cancer and adenocarcinoma cell lines. QMSP and pyrosequencing showed a high rate of concordant results, even if pyrosequencing revealed two different promoter CpGs sub-islands (P1a and P1b) with a different methylation density pattern. Conclusions. Our results confirm the effect of methylation on *KEAP1* transcription control across multiple histologies of lung cancer and suggest pyrosequencing as the best approach to investigate the pattern of CpGs methylation in the promoter region of *KEAP1.* The validation of this approach on lung cancer patient cohorts is mandatory to clarify the prognostic value of the epigenetic deregulation of *KEAP1* in lung tumors.

## 1. Introduction

Cancer cells sustain ROS (Reactive Oxygen Species) production by suppressing the antioxidant-generation system for cellular defense, thereby promoting the effects of oxidative stress and leading to DNA damage and tumor growth [1,2,3]. The KEAP1/NRF2 pathway modulates detoxification processes in normal and neoplastic cells and participates in chemo- and radioresistance of solid tumors. In normal conditions, oxidative and electrophilic changes affect the nuclear factor-erythroid 2-related factor 2 (NRF2) activity by changing the physical interaction with its negative regulator, the Kelch-like ECH-associated protein 1 (KEAP1), thus favoring its ubiquitination and consequent degradation by the 26S proteasome [4,5,6]. In cancer cells, because of chemical modifications of KEAP1 cysteine residues, the inhibition of ubiquitin conjugation to NRF2 by the KEAP1-CUL3 complex leads to NRF2-KEAP1 impairment and results in the nuclear accumulation of the de novo synthesized NRF2 protein [7,8]. The NRF2 overexpression enhances the transcription of the target genes encoding phase II detoxification enzymes and antioxidant proteins [9] and confers chemo- and radio- resistance properties upon cancer cells which become capable of protecting themselves against the surrounding microenvironment and xenobiotics [6]. The *KEAP1* gene spans 17.6 kb of genomic DNA and is located on chr 19:10, 596, 796-10, 614, 417 (GRCh37/hg19) with a minus strand orientation. Two alternatively spliced transcript variants encoding the same protein isoform were annotated for this gene. The first reference transcript (NM_203500) is located at chr19:10596796-10614054 (negative strand, 17259 bp), while the second is mapped (NM_012289) at chr19:10596796-10613481 (16686 bp). Both transcripts encode for a 624 aa protein (Ncbi ID: NP_987096, Uniprot ID: Q14145), [10]. In solid tumors, the accumulation of genetic and epigenetic modifications of *KEAP1* and *NFE2L2* has a critical impact on the regulation of gene expression at transcriptional and post-transcriptional levels [7]. The first report of the loss-of-function mutations of the human *KEAP1* gene was related to NSCLCs with a frequency of 20–25% [11]; these data have been widely confirmed [12]. *KEAP1* mutations commonly occur in the exonic regions that codify for double-glycine repeat/Kelch (DGR) domain that is required for the retention of NRF2 in the cytoplasm. *KEAP1* point mutations were reported in multiple solid cancers with different incidences, such as gastric (11.1%), liver (2–8%), colorectal (7.8%), prostate (1.3%), gallbladder (30.7%), ovarian (37%), glioma (1.7%), head and neck (42%), clear renal cell carcinoma (4.7%) and large cell lung carcinoma (31%), [2,13,14,15,16,17,18,19,20,21,22]. Gain-of-function *NFE2L2* mutations are generally mutually exclusive with *KEAP1*, and often fall into the Neh2 domain (DLG or ETGE motifs), which is the interactive site for KEAP1 binding. *NFE2L2* point mutations were described in esophageal, skin, lung, papillary renal, head and neck and laryngeal carcinomas, and are associated with clinical and prognostic significance [19,21,23,24,25,26].

Aberrant methylation of CpG dinucleotides at the 5′ end of tumor suppressor genes is frequently linked to gene silencing. The *KEAP1* regulatory region included a long CpG-rich island of ~1.2 kb in length (chr19:10613047-10614280) extending from the promoter region to intron 1 within the human hg19/GRCh37 genome sequence. Exonic CpG Island counts 60 CpGs on a total of 397 (chr19:10602281-10602878, hg19/GRCh37) and spans from P2 Region (-88+337) to P1 Region (-291-89), close to the *KEAP1* transcription start site (TSS), [27,28]. In silico analysis demonstrated that functional hypermethylation of CpG-rich sites is mainly assembled within the P1 promoter region that contains 13 different CpGs. This region contains specific consensus protein binding sites, such as GC-box and E-box, as well as AP2-, Sp1-transcription factors, and Ets-binding motifs and their deregulation may play a decisive role in the modulation of KEAP1 expression [29]. *KEAP1* deregulation by CpG hypermethylation appears complex upon investigation; it was studied in large tumor cohorts, but less is known about the details of CpG methylation density pattern [7,29]. Frequent promoter hypermethylation of the *KEAP1* gene and its control role in down-regulation of gene expression was reported by our group in neoplastic tissues of patients affected by glioma, breast cancer (51%), primary NSCLC (47%), and in many cases, it was associated with a worse overall survival in patients [27,28,30,31]. Aberrant *KEAP1* methylation was also reported in 53% of colorectal cancer, head and neck cancer tissues (29.3%) and prostate cancer cells as the main mechanism of epigenetic silencing of *KEAP1* expression with clinical prognostic significance [19,32,33]. Finally, a high frequency of methylation 48.6% was described in clear cell renal carcinoma (ccRC), but a different contribution of CpGs mapped in the promoter region was assessed by The Cancer Genome Atlas (TCGA) data analysis (cg06911149, cg15204119,cg15676203, cg26500801, cg26988016) with a significant association with Overall Survival (OS), grading, staging and tumor dimension [27]. Despite the link between oxidative stress and *KEAP1* has been widely clarified in NSCLC and *KEAP1* mutations, representing one of the main features of these histologies [11,34], a clear association between epigenetic *KEAP1* promoter hypermethylation has not yet been found in NSCLC, and molecular data regarding this deregulation mechanism in lung neuroendocrine tumors are limited [35]. The most frequent approach used to assess *KEAP1* methylation is the quantitative methylation specific PCR (QMSP), [28] but DNA sequencing (i.e., pyrosequencing or bisulfite sequencing) could provide more complete information on the methylation status of the gene promoter in lung tumors.

To address this issue and clarify the role of *KEAP1* promoter hypermethylation in lung tumors, we performed a detailed scanning of each single CpG within *KEAP1* promoter of cell lines from different lung tumor histologies. We used two different methodological approaches of QMSP and pyrosequencing, and compared the obtained results. The impact of *KEAP1* methylation on its transcriptional activity was also assessed by in vitro demethylating treatments in SCLC, carcinoid and NSCLC cells.

## 2. Results

### 2.1. KEAP1 Promoter Methylation Patterns among Different Lung Cancer Histologies

**Figure 1:**
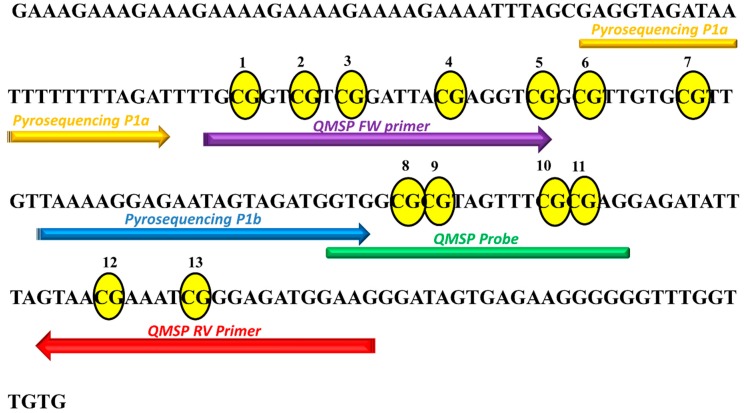
Sequence of *KEAP1* P1 promoter region (-291 -89 bp from the TSS, transcription starting site) scanned by QMSP and pyrosequencing. CpGs are marked as yellow circles and numbers are given to the CpGs from 5′ to 3′ of positive strand of the *KEAP1* gene. The primers used for QMSP and pyrosequencing analysis are indicated.

Methylation analysis of the *KEAP1* promoter region was performed by two different detection methods: QMSP and pyrosequencing. Both methodologies were used to profile the same P1 promoter *KEAP1* region containing 13 CpG sites (Figure 1) [7]. A total of 25 cell lines from different histologies of lung cancers were screened: SCLC, Atypical and Typical Carcinoid, ADC, SqCC and LCC. Two cell lines from normal lung tissues (MRC5 and BEAS-2B) were also analyzed to establish the cut-off value of methylation to use for QMSP and pyrosequencing data scoring.

**Figure 2:**
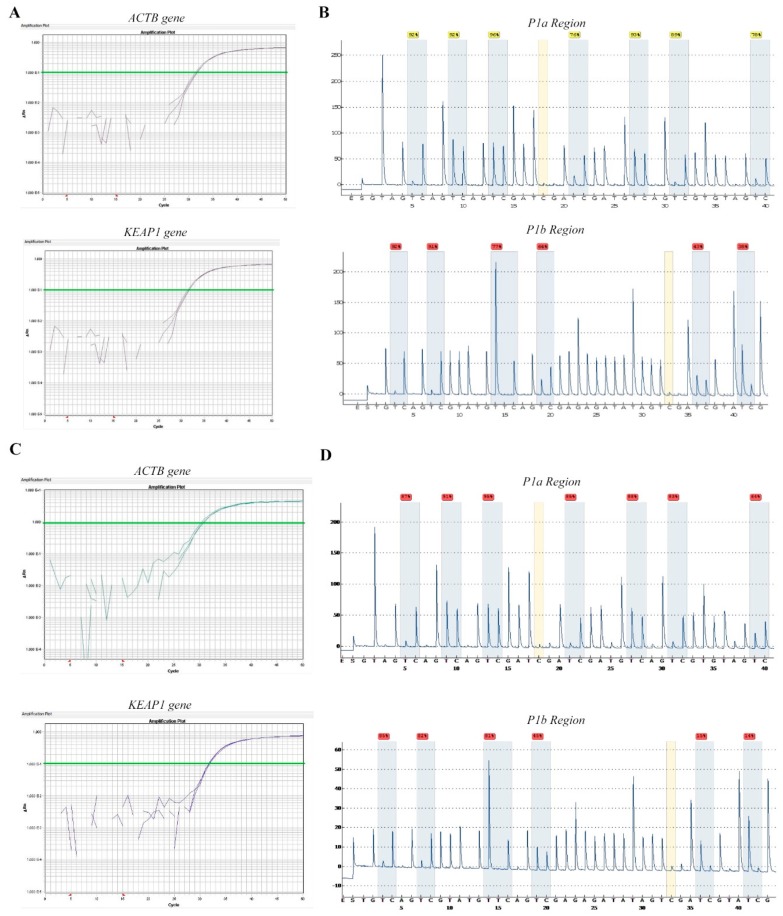
Amplification panels by QMSP and pyrograms of *KEAP1* hypermethylated H69V and H1573 cells. Amplification curves (Ct values versus ΔRn) of *ACTB* and *KEAP1* genes were reported for (**A**) H69V and (**C**) H1573. Representative *KEAP1* promoter pyrograms after DNA bisulfite conversion obtained with the first (P1a region) and second (P1b region) reactions of primers (1–7 and 8–13 CpG sites, respectively) were reported for (**B**) H69V and (**D**) H1573 cell lines.

For QMSP data evaluation, the cut-off level of methylation analysis established on normal cell lines was set at 0. *KEAP1* methylation levels measured in the tumor cell lines ranged as follows: 0–228 (SCLC), 0–78.8 (Carcinoids), 0–492 (ADC, SqCC, LCC). Based on the cut-off level, a total of 5/12 (34%) of SCLC cell lines, (GLC8, H1963, H209, H69V), 1/2 (50%) of carcinoid cell lines (H720) was scored as hypermethylated (Table 1). Similarly, *KEAP1* hypermethylation was observed in half of total cell lines analyzed with NSCLC histologies. Specifically, it was detected in 4/8 (50%) of ADCs (A549, H1573, H1395, H1581), 1/2 (50%) of SqCCs (HCC-15) and in the LCC cell line H460, (Figure 2).

The results from methylation analysis of *KEAP1* promoter region by QMSP and pyrosequencing are shown in Table 1. A total of 4 out of 12 SCLC cell lines (34%) were positive for *KEAP1* methylation, with mean values of 78.5% (H69V), 27.4% (H209), 39.5% (H1963) and 35.5% (GLC8). H720 carcinoid cells appeared methylated (67.8%), in contrast to the H727, which was under the analytical cut-off. In NSCLC cell lines, hypermethylation at the P1 promoter region was found in 4/8 (50%) of cell lines: A549, H1573, H1395 (ADC), HCC-15 (SqCC) and H460 (LCC), with the H1573 as the most methylated one (70.4%). These QMSP data seem to be almost comparable with those from pyrosequencing among different lung cancer histologies, and in many cases share the same distribution and density pattern for the 13 CpG sites. Universal Methylated Human DNA bisulfite-converted showed a reliability of about 96%, with high coverage in all CpG sites mapped into the promoter region examined, whereas the Universal Unmethylated Human DNA, used as negative control, showed a mean of methylation levels of 3% (Supplemental Figure S1). The mean cut-off of 26.43 for *KEAP1* methylation by pyrosequencing was determined using the normal lung cell lines MRC5 and BEAS-2B as the mean of methylation values of the 13 CpG sites of the P1 region. The cut-off at single CpG sites was also calculated as indicated: 74.1% (PYRCpG1), 36.2% (PYRCpG2), 41.5% (PYRCpG3), 20.1% (PYRCpG4), 79.0% (PYRCpG5), 72.0% (PYRCpG6), 23.2% (PYRCpG7), 25.6% (PYRCpG8), 22.6% (PYRCpG9), 14.7% (PYRCpG10), 12.6% (PYRCpG11), 6.4% (PYRCpG12), 6.3% (PYRCpG13), (Supplemental Table S1A,B).

### 2.2. Technical Evaluation of Pyrosequencing

The two cell lines MRC5 and BEAS-2B were used to establish the intra- and inter-assay precision of pyrosequencing analysis to detect methylation at *KEAP1* P1 promoter region. Both cell lines were found to be unmethylated by QMSP analysis. Each cell line was tested 3 times in five different runs on separate plates to assess the inter-assay precision, whereas to test the intra-assay precision, the same cell lines were replicated 3 times in a single run. The mean values of each experiment were used to calculate the inter-assay coefficient of variation (CV). The inter-assay CV ranges from 11.6% and 8.1% for MRC5 and BEAS-2B, respectively (Table 2A). The intra-assay CV was very similar between MRC5 (6.6%) and BEAS-2B (6.9%), (Table 2B). To define the precision of the technique to quantify the methylation status at each single CpG of P1 region, CV values for inter and intra-assay were also calculated for the methylation values of the 13 CpGs included into the pyrosequencing analysis, both for MRC5 and BEAS-2B cells (Table 3A,B).

### 2.3. Pyrosequencing Analysis Reveals Two Distinct P1 Subregions at KEAP1 Promoter

**Figure 3:**
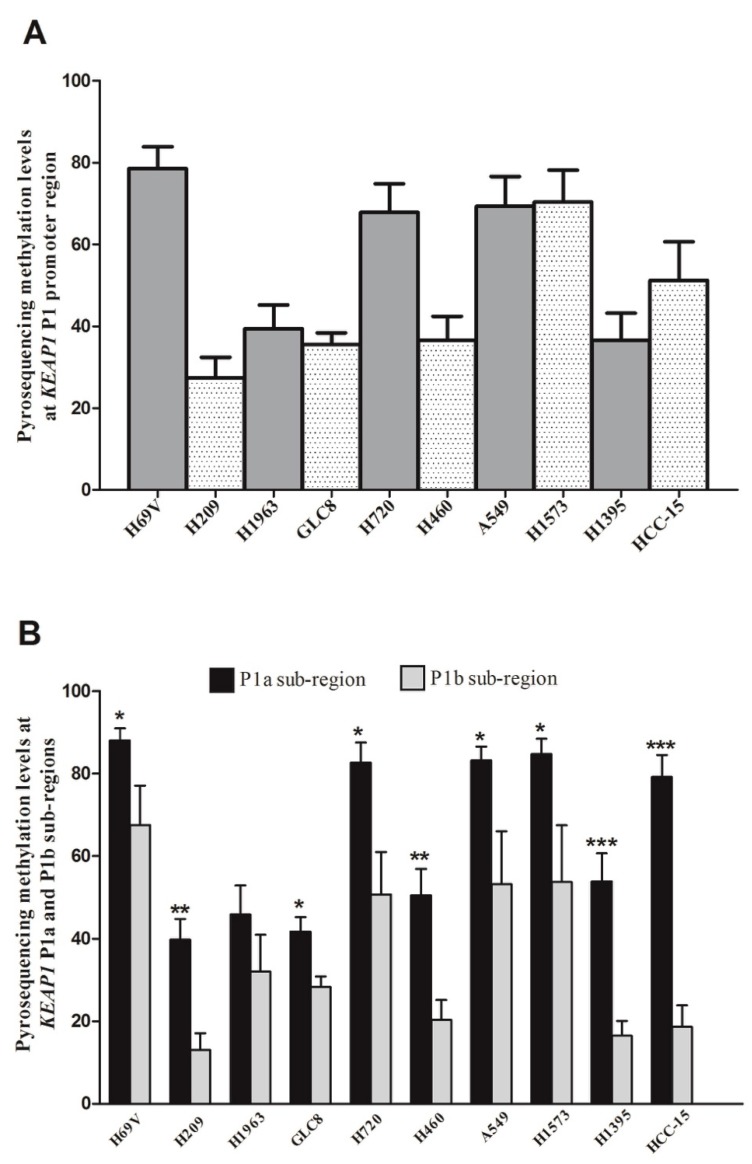
(**A**) Histograms showing the methylation levels detected by pyrosequencing at *KEAP1* P1 promoter region in positive lung cancer cell lines. (**B**) Pyrosequencing methylation levels at P1a and P1b sub-regions of *KEAP1* promoter in methylated lung cancer cell lines. *p* value was set at * *p* < 0.05; ** *p* < 0.01; *** *p* < 0.001, *t*-test.

Despite the high concordant results between QMSP and pyrosequencing, an interesting dual pattern of methylation at *KEAP1* P1 promoter region was revealed by pyrosequencing. The *KEAP1* region containing 1−7 CpGs (P1a sub-region) showed significant higher methylation levels than the promoter region which contains 8−13 CpGs (P1b sub-region), (Figure 3). In light of these results, the first seven single CpG sites appeared to be more methylated than the others, and could represent the critical sub-region closer to the transcription start site that exerts a stronger regulation impact on the *KEAP1* promoter region and its transcript levels.

### 2.4. Somatic Alterations of KEAP1 Detected in Lung Cancer Cell Lines

Loss-of-function point mutations of the *KEAP1* gene were identified in seven cell lines (Table 4). These missense mutations are known to have a pathogenic significance, except for the de novo nucleotidic c.269C > T and aminoacidic change p.A90V that remain to be characterized by functional studies. These genetic findings confirm the already published evidence that missense mutations impact the efficiency of *KEAP1* stability and its ability to bind NRF2 transcription factor, thus contributing to KEAP1-mediated repression of NRF2 with its consequent nuclear accumulation [7]. By contrast, no mutation was found in the *NFE2L2* gene. In general, the rarity of point mutations to KEAP1/NRF2 pathway deregulation were confirmed in SCLC cells, whereas the high frequency of missense mutations in the subset of NSCLC cells represent a molecular and distinctive hallmark [11,21].

### 2.5. The Restoration of KEAP1 Expression Via Its Promoter P1 Region Inversely Correlates with Demethylation by 5-aza-dC

**Figure 4:**
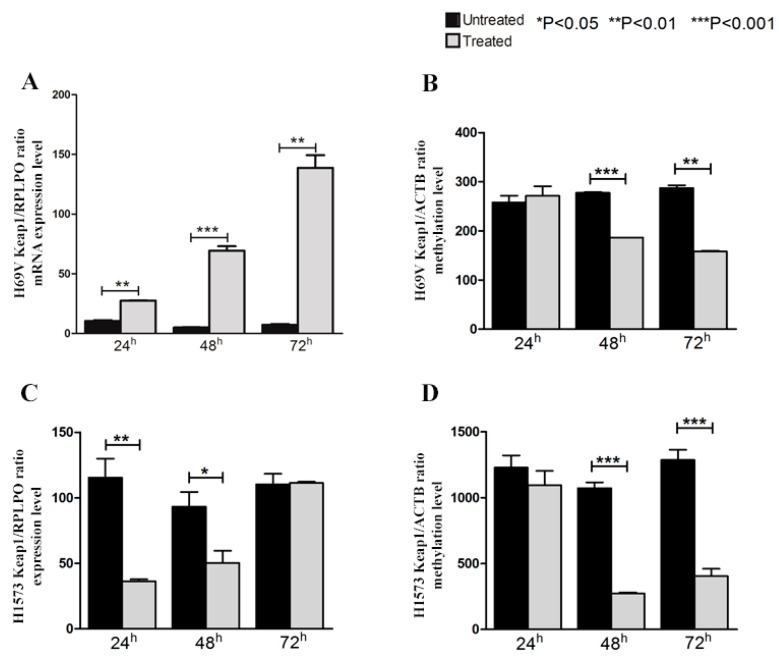
Changes in *KEAP1* mRNA transcript levels in the (**A**) H69V, (**C**) H1573 cell lines by RT-PCR before (CTRL) and after treatment with 5 μM of 5-aza-dC at 24 h, 48 h, 72 h. Error bars indicate the standard deviation of three different experiments. Changes in *KEAP1* promoter methylation levels in the (**B**) H69V, (**D**) H1573 cell lines by QMSP before (CTRL) and after treatment with 5 μM of 5-aza-dC at 24 h, 48 h, 72 h. Error bars indicate the standard deviation of three different experiments. * *p* < 0.05, ** *p* < 0.01, *** *p* < 0.001.

A direct correlation of *KEAP1* promoter methylation with mRNA levels was also confirmed by in vitro 5-aza-dC treatment for H69V, H1573 and H720 cells. The variation of *KEAP1* mRNA levels and promoter methylation levels in H69V and H1573 (Figure 4A,C) cell lines before and during treatment with 5-aza-dC. By real-time quantitative PCR analysis, a progressive increase in the *KEAP1* transcript abundance was observed after 48 h (*p* < 0.05; *p* < 0.001, for H69V and H1573 respectively) and 72 h (*p* < 0.01 only for H69V) and was shown to correlate with a decreased *KEAP1* promoter methylation at 48 h (both with *p* < 0.001) and at 72 h (*p* < 0.01; *p* < 0.001, for H69V and H1573 respectively), (Figure 4B,D).

## 3. Discussion

The epigenetic control of *KEAP1* expression by methylation has been widely reported in solid tumors, and represents in many contexts a multifaceted prognostic marker for disease outcome [7]. In glioma patients, the co-occurrent hypermethylation of *KEAP1* and *MGMT* predicts a lower risk of progression for patients treated with radiotherapy and temozolomide [28]. In triple-negative breast cancer patients with *KEAP1* methylation, a higher mortality risk was observed than in patients without triple-negative breast cancer. By contrast, the *KEAP1* methylation was associated with a better progression free survival in patients treated with epirubicin/cyclophosfamide and docetaxel as sequential chemotherapy [30]. Aberrant *KEAP1* methylation was also reported in colorectal cancer and head and neck cancer tissues, and was linked to the worst prognoses of these tumors [32,36]. In clear renal cell carcinoma (ccRCC), the TCGA data analysis suggested that epigenetic silencing by methylation is able to strongly predict patient survival [27].

Despite the well-documented impact of *KEAP1* and *NFE2L2* mutations in NSCLCs and LCNEC [34], the prognostic role of *KEAP1* methylation in lung cancer has not yet been clarified. The selective inhibition of *KEAP1* gene promoter methylation by genistein, observed in A549 cells, suggested a way in which *KEAP1* demethylation could represent a marker of radio-sensitizing effects in lung cancer [37]. In lung cancer tissues, the presence of epigenetic abnormalities in the *KEAP1* gene plus its point mutations/LOH matched with the prevalence of NRF2 nuclear accumulation in NSCLC tissues and was associated with an increased risk of lung cancer progression in surgically resected patients [31]. By contrast, in NSCLCs tissues, the *KEAP1* methylation alone assessed by QMSP does not seem to be an independent prognostic marker of disease outcome.

Our work aimed first to assess the methylation pattern of KEAP1 promoter region in different histotypes of lung cancer cell lines by performing a QMSP vs pyrosequencing evaluation for the first time. *KEAP1* hypermethylation was detected in 50% of NSCLC cell lines (both ADCs and SqCCs), in atypical carcinoids and described for the first time in 42% of SCLC cell lines. The latter result is not yet reported and was surprising, since it gives the first indication of epigenetic lesions of *KEAP1* gene in the SCLC. Even if few important indications of *KEAP1* genetic alterations in high grade neuroendocrine lung tumors (LCNEC) with adeno-like features came from two recent works [35,38], SCLC point mutations of *KEAP1* and *NFE2L2* genes remain a rare phenomenon, and the epigenetic modulation of *KEAP1* expression has not yet been elucidated.

Highly variable levels of *KEAP1* promoter methylation by QMSP were observed up to the cut-off level in all lung cell line, independently from the histology with no linear correlation between the QMSP and pyrosequencing values was observed (*p* = 0.19, for Pearson correlation). However, it should be noted that samples what were methylated above the QMSP and pyrosequencing cut-off were almost completely overlapping, and only in one case (H1581 cell line) was the result not concordant.

Currently, there is no consensus on the best technique for *KEAP1* methylation assessment and how many and which CpG sites of *KEAP1* promoters should be analyzed remains a controversial issue in a translational context [7]. There are several molecular diagnostic tools that can be used to assess the methylation status to influence single-nucleotide resolution information about the methylated areas of DNA after bisulfite treatment and translate these findings into clinical setting [39]. QMSP amplifies methylated DNA and quantifies a target methylation level relative to house-keeping genes (e.g., b-actin). Pyrosequencing, on the other hand, amplifies bisulfite converted genomic DNA using primers independent of methylation status, and quantifies the percentage of methylated CpGs with single base resolution. Both methods represent simple, fast, and cost-effective techniques that can be easily implemented into clinical practice; however, studying methylation of each CpG separately should unmaske putative differences of methylation pattern in lung cancer with different histologies that would explain the absence of correlation between QMSP levels and mean of CpG methylation of the same region of the same samples status by pyrosequencing.

Most interestingly, our analysis of the pattern and density of *KEAP1* promoter methylation in lung cancer cell lines showed that the first seven single CpG sites (1–7, P1a region) appeared to be significantly more methylated than the last six CpG group (8–13, P1b region) of the P1 promoter region. Based on these results, a stronger effect and impact on *KEAP1* expression level should be hypothesized by the CpG sites 1-7 located in the critical sub-region closer to the transcription start site (TSS) of gene (P1a). An intriguing possible explanation is that in the P1a region two putative binding sites for Sp1 and AP2 were mapped [29,40], two zinc finger transcription factors that contribute to the crucial transcriptional activity of this gene [41]. As a consequence, we supposed that the hypermethylation of the P1 *KEAP1* promoter might lead to the loss of SP-1 and AP-2 binding via inhibiting its expression at the first two, 4^th^ and 5^th^ CpG sites in the analyzed lung cancer cells, respectively [29,40].

In this study we also confirmed that epigenetic silencing of *KEAP1* by promoter methylation exerts a critical role in the modulation of *KEAP1* transcription activity. An inverse correlation between *KEAP1* mRNA levels and methylation levels was demonstrated by in vitro 5′-azacytidine treatment on carcinoid, SCLC and ADC cells, thus corroborating the general idea that consensus sequences of several transcription sites was marked in the epigenetic control of *KEAP1*.

This analysis opens the debate on how many and which CpG sites of the *KEAP1* promoter should be analyzed to set a clinical cut-off in lung cancer affected patients. Further prospective analyses in lung cancer tissues are ongoing to define whether some specific CpG sites of *KEAP1*, either a combination of them even if not consecutive, might have a better predictive or prognostic role in lung cancer patients.

## 4. Material and Methods

### 4.1. Cell Lines

A set of 25 lung cell lines were used, histologically classified as follows: 12 SCLC cell lines (H69V, H209, H1184, N417, H2107, H1963, GLC1, GLC2, GLC8, GLC14, H510, H2141), 1 typical carcinoids cell line (H727), 1 atypical carcinoid cell line (H720), 8 ADC (H1581,H2126, A549, H1573, H2228, H1975, HCC4006, H1395), 2 SqCC cell lines (HCC-15, H520) and 1 LCC cell line (H460). GLC1, GLC2, GLC8, GLC14 cell lines were kindly provided by Dr. Clelia Tiziana Storlazzi (University of Bari, Italy). All the other cell lines were purchased from the American Type Culture Collection (ATCC, Manassas, Virginia, United States). The two normal lung fibroblast and epithelial MRC5, BEAS-2B cell lines were used as controls. Cells were cultured in RPMI 1640 medium supplemented with 10% or 20% fetal bovine serum (FBS), 100 U/mL penicillin and 100 U/mL streptomycin, and maintained at 37 °C in a 5% CO_2_ incubator. Cell culture reagents were purchased from Euroclone (Euroclone, Milan, Italy).

### 4.2. DNA and RNA Extraction from Cell Lines

Cells from 1 well of 12-multiwell were extracted by using the standard Phenol/chloroform procedure. DNA pellets were suspended in LOTE solution (3 mM Tris-HCl (pH 8.0)/0.2 mM EDTA, pH 8.0) and estimated by NanoDrop Spectrophotometer ND-1000 (Thermo Scientific, Carlsbad, CA, USA). RNA was extracted using Trizol reagent (Life Technologies) according to the manufacturer’s instructions. The RNA quality was measured by using 2100 Expert Analyzer (Agilent Technologies, Santa Clara, CA, USA) and RNA with RIN (RNA Integrity Number) ≥7.0 was processed. RNA concentrations were quantified by the Nanodrop spectrophotometer.

### 4.3. DNA Sodium Bisulfite Conversion and Quantitative Methylation Specific PCR (QMSP)

Bisulfite conversion and purification of DNA extracted from cell lines was performed by Epitect Bisulfite kit (QiagenSci, MD, USA) according to manufacturer’s instruction. Bisulfite-modified DNA was then used as template for QMSP analysis and calibration curves for both target and reference genes were constructed using serial dilutions (90–0.009 ng) of commercially available fully methylated DNA (CpGenome Universal Methylated DNA, Millipore, Chemicon, cat#S7821, Bedford, MA, USA). Amplification reactions were carried out in triplicate in 384-well plates and in a volume of 10 uL that contained 50 ng of bisulfite-modified DNA on an ABI PRISM 7900 Sequence detection system and were analyzed by SDS 2.4.1 software (Thermo Fisher Inc., Applied Biosystems Division). PCR primers were used in real-time PCR amplified the *KEAP1* promoter region of 64bp and was just reported in several methylation studies of *KEAP1*: forward 5′- TGCGGTCGTCGGATTACGAGGTCG-3′, reverse 5′-CTTCCATCTCCCGATTTCGTTAC-3′ and probe FAM-GTGGCGCGTAGTTTCGCGAG-TAMRA. As reference gene, a primer/probe set specific for the unmethylated promoter region of the *ACTB* gene was used: forward 5′-TGGTGATGGAGGAGGTTTAGTAAGT-3′, reverse 5′-AACCAATAAAACCTACTCCTCCCTTAA-3′ and probe FAM-ACCACCACCCAACACACAATAACAAACACA-TAMRA [27,28]. Each plate included calibration curves for the *ACTB* and *KEAP1* genes, DNA cell lines, a positive control CpGenome Universal Methylated DNA, and multiple water blanks. The QMSP standard curves of the *KEAP1* and *ACTB* genes for the normalization of the input DNA were established with CpGenome Universal Methylated DNA. The relative level of methylated DNA was finally calculated as the ratio of *KEAP1* to *ACTB* and then multiplied by 1000 for easier tabulation (average value of triplicates of *KEAP1*/average value of triplicates of *ACTB* × 1000).

### 4.4. Pyrosequencing

Pyrosequencing was performed on PyroMark Q24 (Qiagen, Hilden, Germany) and amplifications were carried using 50ng of bisulfite-treated genomic DNA out in 24-well plates according to the manufacturer instructions. Amplification primers used for the *KEAP1* promoter region were previously described and are as follows: forward: 5′-GTTTGAGGTTAGGAGTTTAAGGTTG-3′, reverse: 5′-CACAACCAAACCCCCCTT-3′. The reverse primer contained biotin at the 5′ position [37]. These two assays were designed and run on this template using two sequencing primers: 5′-GAGGTAGATGATTTTTTTTAGAT-3′ (assay for CpGs 1-7) and TAAAAGGAGAATAGTAGATGGTG (assay for CpGs 8–13). For the pyrosequencing reaction, single-stranded DNA templates were immobilized on streptavidin-coated sepharose beads (Qiagen, Hilden, Germany) using the PSQ Vacuum Prep Tool and Vacuum Prep Worktable (Qiagen, Hilden, Germany), then incubated at 80 °C for 2′. In addition to the samples, each run included a non-template control (water), the unmethylated control and the methylated control. The dispensation order was (two dispensations added for each *KEAP1* sequencing primers). The two dispensation orders were automatically generated by PyroMark Q24 Software 2.0.8 according to the provider recommendations were: GTAGTCAGTCAGTCGATCGATCGATGTCAGTCGTGTAGTC for the first *KEAP1* sequencing primer flanking the 1-7 CpG sites (P1a region) and TGTCAGTCGTATGTTCAGTCGAGAGATATAGTCGATCGTATCG for the second *KEAP1* sequencing primer flanking the 8-13 CpG sites (P1b region). The proportion of DNA methylation at each CpG site was automatically calculated by the abovementioned PyroMark Q24 Software and given as a percentage. The % methylated fraction (C/T ratio) was displayed in a small colored box just above each CpG site in the analyzed sequence [37]. For each CpG island tested, a mean result was calculated. For data analysis, the average percentages of all 13 CpGs (whole P1 region), 1-7 CpGs (P1a region) and 8-13 CpGs (P1b region) were determined as well as the results of each tested CpG.

### 4.5. Pyrosequencing Intra and Inter-Assay Analysis

To assess the intra-assay precision of pyrosequencing, two cell lines (MRC5 and BEAS-2B) were tested 3 times in a single run. To evaluate the inter-assay variation, the same cell lines were tested 5 times in 3 different runs. To obtain the mean and standard deviation values from each control, the coefficient of variation (CV) was used to assess the pyrosequencing performance by determining the ratio by the standard deviation and mean for both inter- and intra-assay.

### 4.6. Mutation Profiling of Cell Lines

Exon/intron gene structure were obtained from NCBI/Genbank databases and primers set used for genetic screening were designed in order to cover the entire region of the DGR domain of the *KEAP1* (exons 4–6) and the exon 2 of *NFE2LE* gene [27]. PCR amplification of each fragment was performed by using Gene Amp PCR System 9700 thermal cycler (Applied Biosystem, Foster City, CA, USA). PCR products were purified using GFX PCR DNA and the Gel Band Purification Kit (GE Healthcare, Buckinghamshire, UK) and sequenced by using the Big Dye Terminator Ready Reaction mix v. 1.1 on an ABI 3100 sequence detection system with the Sequencing Analysis software v.3.7 (Applied Biosystems), [31].

### 4.7. Quantification of KEAP1 Expression by Real-Time Quantitative PCR Analysis

The StrataClonePCR Cloning Vector (Stratagene, Milan, Italy) was used to clone PCR fragments for *KEAP1* and *RPLPO* genes which were amplified by TaqMan assays (*KEAP1* Assay ID: Hs00202227_m1, Applied Biosystems and *RPLPO* Assay ID: 4326314E, Thermo Fisher). We constructed different standard curves for *RPLPO* and *KEAP1* genes with different copy numbers of plasmids. SuperScript III First-Strand Synthesis (Thermo Fisher, Invitrogen Division, Carlsbad, CA, USA) was used to obtain cDNA synthesis from 1 μg of total RNA extracted from cell lines. Detection by real-time RT-PCR was performed with TaqMan Gene Expression Assays (Thermo Fisher, Life Technologies division). The reactions were run in triplicate on an ABI PRISM 7900HT Sequence Detection System (Thermo Fisher, Life Technologies Division). mRNA levels of *KEAP1* were calculated by normalizing its calibration curves and its sample concentration versus *RPLPO* (endogenous gene). The relative sample amount that results in plasmid concentrations expressed as copy number of corresponding standard molecules was calculated according the following formula: Target/Housekeeping*1000.

### 4.8. In vitro 5-Aza-2′-deoxycytidine (5-aza-dC) Treatment

H69V, H1573 and H720 cell lines were seeded in a six-well dish. The 5-aza-2′-deoxycytidine, an inhibitor of DNA methyltransferase, was added in a concentration of 5 μM (Sigma-Aldrich, St. Louis, MO, USA) with fresh medium for 24 h, 48 h, and 72 h in each one of them. At each time points cells were harvested for DNA and RNA isolation to measure DNA methylation at *KEAP1* promoter region by QMSP and KEAP1 transcription levels.

### 4.9. Statistical Analysis

Data are expressed as mean ± SEM of the number of in vitro experiments (*n*) indicated in the figure legends. In all the assays “*n*” is referred to the number of independent experiments performed on different cell preparations. For in vitro experiments, at least three to seven different wells were analyzed. All statistically significant differences were computed using a t-test, the significance level being set at * *p* < 0.05; ** *p* < 0.01; *** *p* < 0.001. All graphs shown, were performed by GraphPad Prism 5.

## 5. Conclusions

Our results confirm the effect of methylation on *KEAP1* transcription control across multiple histologies of lung cancer. Since QMSP does not provide methylation frequency at individual CpG sites, we suggest pyrosequencing as the best approach to investigate the pattern of CpGs methylation in the promoter region of *KEAP1.* The validation of this approach on lung cancer patient cohorts is essential to clarify the prognostic value of the epigenetic deregulation of *KEAP1* in lung tumors.

## Figures and Tables

**Table 1:** *KEAP1* P1 promoter methylation levels assessed by QMSP and pyrosequencing.

Lung Cancer Cells	Histology	QMSP	CpG1	CpG2	CpG3	CpG4	CpG5	CpG6	CpG7	CpG8	CpG9	CpG10	CpG11	CpG12	CpG13	Pyrosequencing (Mean % ± SD) *	P1 Region Mean (%) ± SD **	P1a Region Mean (%) ± SD **	P1b Region Mean (%) ± SD **
H69V	SCLC	138	92	92	96	76	93	89	78	92	91	77	64	43	38	78.5 ± 19.3	79 ± 20	88 ± 8	68 ± 23
H209	SCLC	4.8	55	47	54	23	40	37	22	21	28	15	7	4	3	27.4 ± 18.0	27 ± 13	40 ± 13	13 ± 10
H1184	SCLC	0	14	9	13	4	7	5	6	8	9	8	2	2	2	6.9 ± 3.9	7 ± 5	8 ± 4	5 ± 3
N417	SCLC	0	21	19	19	7	11	15	10	12	12	6	3	1	2	10.6 ± 6.7	11 ± 6	15 ± 5	6 ± 5
H2107	SCLC	0	20	24	24	6	18	10	10	7	8	8	3	1	2	10.8 ± 8.0	11 ± 5	16 ± 7	5 ± 3
H1963	SCLC	15	61	57	68	13	46	40	36	56	51	44	28	7	6	39.5 ± 20.6	39 ± 32	46 ± 19	32 ± 22
GLC1	SCLC	0	21	25	24	5	12	10	9	19	15	8	3	1	2	11.8 ± 8.3	12 ± 8	15 ± 8	8 ± 7
GLC2	SCLC	0	26	24	21	4	10	13	8	17	13	9	5	2	2	11.8 ± 8.1	12 ± 8	15 ± 9	8 ± 6
GLC8	SCLC	228	54	46	53	31	38	37	33	34	35	32	26	21	22	35.5 ± 10.4	36 ± 28	42 ± 9	28 ± 6
GLC14	SCLC	0	22	15	17	5	10	10	8	9	9	5	2	1	2	8.8 ± 6.2	9 ± 5	12 ± 6	5 ± 4
H510	SCLC	0	8	4	7	1	3	3	3	2	3	4	1	0	1	3.1 ± 2.3	3 ± 2	4 ± 2	2 ± 1
H2141	SCLC	0	17	12	14	4	7	9	6	6	7	6	2	2	2	7.2 ± 4.7	7 ± 4	10 ± 5	4 ± 2
H720	AC	78.8	92	88	96	61	89	84	68	77	74	65	42	15	31	67.8 ± 25.0	68 ± 51	83 ± 13	51 ± 25
H727	TC	0	21	14	21	7	11	11	8	7	7	11	3	2	5	9.8 ± 6.0	10 ± 6	13 ± 6	6 ± 3
H460	LCC	66	65	64	72	28	50	41	33	23	32	33	9	4	21	36.5 ± 21.2	37 ± 20	50 ± 17	20 ± 12
H2126	ADC	0	68	58	65	15	29	27	18	20	17	16	5	3	8	21.4 ± 14.4	27 ± 12	40 ± 23	12 ± 7
A549	ADC	492	88	88	95	73	87	81	70	87	84	69	43	18	18	26.8 ± 22.4	69 ± 53	83 ± 9	53 ± 31
H1573	ADC	123	87	91	95	85	88	83	64	85	82	81	45	15	14	69.3 ± 26.3	70 ± 54	85 ± 10	54 ± 34
H2228	ADC	0	43	47	37	13	19	24	16	13	25	12	9	3	6	70.4 ± 28.0	21 ± 11	28 ± 14	11 ± 8
H1975	ADC	0	16	10	14	6	10	1	8	4	4	9	2	2	4	20.5 ± 14.1	7 ± 4	9 ± 5	4 ± 3
HCC4006	ADC	0	40	35	48	11	24	17	15	6	19	23	4	2	20	6.9 ± 4.7	20 ± 12	27 ± 14	12 ± 9
H1395	ADC	17	72	74	68	36	56	36	35	24	26	23	11	5	10	20.3 ± 13.9	37 ± 17	54 ± 18	17 ± 9
H1581	ADC	30	44	36	38	12	26	31	5	30	27	16	8	2	3	36.6 ± 23.9	21 ± 14	27 ± 14	14 ± 12
HCC-15	SqCC	12	85	87	94	54	89	78	67	25	38	24	7	5	13	51.2 ± 33.9	51 ± 19	79 ± 14	19 ± 13
H520	SqCC	0	45	34	41	12	25	23	14	14	18	14	5	2	8	19.6 ± 13.4	20 ± 10	28 ± 13	10 ± 6

QMSP, quantitative methylation specific PCR; SD, standard deviation. P1a sub-region from 1 to 7 CpGs; P1b sub-region from 8 to 13 CpGs. SCLC, small cell lung cancer; AC, atypical carcinoid; TC, typical carcinoid; LCC, large cell carcinoma; ADC, adenocarcinoma; SqCC, Squamous Cell Carcinoma. * methylation levels were reported as mean ± SD of methylation levels of total 13 CpGs mapped at the *KEAP1* P1 promoter region. ** methylation levels by pyrosequencing for P1, P1a and P1b were reported as mean ± SD of methylation levels of single CpG mapped at each specific region. The value of methylation of each CpG site is reported and is expressed as a percentage (%).

**Table 2:** (**A**) Inter-assay precision for *KEAP1* P1 promoter methylation analysis by pyrosequencing. (whole P1 region). (**B**) Intra-assay precision for *KEAP1* P1 promoter methylation analysis by pyrosequencing (whole P1 region).

**(A)**								
**Normal Cell Lines**	**RN1 Mean ± SD**	**RN2 Mean ± SD**	**RN3 Mean ± SD**	**RN4 Mean ± SD**	**RN5 Mean ± SD**	**ALL RPs Mean ± SD**	**SD**	**CV%**
MRC5	19.8 ± 12.4	16 ± 10.4	20.2 ± 12.3	22.3 ± 9.6	19.7 ± 11.9	19.6 ± 1.2	1.2	6.1
BEAS-2B	24.8 ± 21.6	24.8 ± 18.2	23.2 ± 19.5	20.3 ± 17.5	22.5 ± 20.8	23.1 ± 1.7	1.7	7.4
**(B)**								
**Normal Cell Lines**	**RP1 Mean ± SD**	**RP2 Mean ± SD**		**RP3 Mean ± SD**		**ALL RPs Mean ± SD**	**SD**	**CV%**
MRC5	20.2 ± 12.3	22.3 ± 9.6		19.7 ± 11.9		20.7 ± 1.4	1.4	6.8
BEAS-2B	23.2 ± 19.5	20.3 ± 17.5		22.5 ± 20.8		22 ± 1.7	1.7	7.7

(**A**) CV, coefficient of variation; SD, standard deviation; RN, run. To test inter-assay precision of pyrosequencing, two cell lines (MRC5 and BEAS-2B) were tested in 5 different runs (RNs). Values of methylation were reported as means of methylation levels of all 13 CpGs. Each value is expressed as a percentage (%). (**B**) CV, coefficient of variation; SD, standard deviation; RP, repetition. To test intra-assay precision of pyrosequencing, two cell lines (MRC5 and BEAS-2B) were tested 3× in the same run (RPs). Methylation level was reported as the mean of methylation levels of 13 CpGs. Each value is expressed as a percentage (%).

**Table 3:** (**A**) Inter-assay precision for *KEAP1* P1 promoter methylation analysis by pyrosequencing (single CpG). (**B**) Intra-assay precision for *KEAP1* P1 promoter methylation analysis by pyrosequencing (single CpG).

**(A)**			
**CpG**	**MRC5 (Mean ± SD)**	**BEAS-2B (Mean ± SD)**	**CV %**
1	37 ± 3.7	56.4 ± 6.1	2.6
2	34.6 ± 4.3	32.8 ± 4.7	0.6
3	39.2 ± 5.2	40.4 ± 3.1	2.6
4	17 ± 1.4	13.6 ± 2.1	2.2
5	22 ± 5.7	51.8 ± 5.7	0.1
6	23.8 ± 4.0	49 ± 2.2	2.4
7	17.8 ± 1.6	20.6 ± 4.2	6.6
8	17.6 ± 5.8	8.8 ± 1.1	17.7
9	16.4 ± 2.7	9.6 ±1.9	2.9
10	11.2 ± 2.9	7.4 ± 4.4	8.2
11	8.8 ± 2.0	4.6 ± 1.5	4.0
12	4.6 ± 3.0	2.6 ± 1.5	21.3
13	4.8 ± 2.5	3.2 ± 2.7	2.4
**(B)**			
**CpG**	**MRC5 (Mean ± SD)**	**BEAS-2B (Mean ± SD)**	**CV %**
**1**	37.3 ± 2.5	54.3 ± 7.5	5.4
**2**	36.7 ± 1.2	29.7 ± 1.2	0.0
**3**	40.0 ± 1.7	40.0 ± 4.4	3.3
**4**	17.0 ± 2.0	12.3 ± 0.6	4.8
**5**	24.7 ± 1.2	49.3 ± 4.7	4.8
**6**	25.3 ± 1.5	50.0 ± 2.0	0.6
**7**	17.3 ±2.1	18.3 ± 3.8	4.8
**8**	20.7 ± 5.5	8.3 ± 1.2	15.0
**9**	17.7 ± 1.5	9.0 ± 2.0	1.8
**10**	12.3 ± 2.3	6.7 ± 4.0	9.1
**11**	9.3 ± 2.5	4.3 ± 2.1	3.2
**12**	5.7 ± 3.8	2.0 ± 1.0	36.3
**13**	5.7 ± 3.1	2.0 ± 0.0	39.8

(**A**) CV, coefficient of variation; SD, standard deviation. To test inter-assay precision of pyrosequencing, two cell lines (MRC5 and BEAS-2B) were tested in 5 different runs. Each value is expressed as a percentage (%). (**B**) CV, coefficient of variation; SD, standard deviation. To test intra-assay precision of pyrosequencing, two cell lines (MRC5 and BEAS-2B) were tested 3× in the same run (RPs). Each value is expressed as a percentage (%).

**Table 4:** Genetic alterations of *KEAP1* gene identified in lung cell lines.

Cell Line	Histology	Gene	Protein Domain	Nucleotidic Change	Amino Acid Change
H1184	SCLC	*KEAP1*	KELCH2	c.1090G > T	p.G364C
H460	LCC	*KEAP1*	IVR	c.706G > C	p.D236H
H2126	ADC	*KEAP1*	IVR	c.814C > T	p.R272C
A549	ADC	*KEAP1*	KELCH1	c.997G > T	p.G333C
H1573	ADC	*KEAP1*	KELCH3	c.1238G > T	p.L413R
H2228	ADC	*KEAP1*	BTB	c.269C > T	p.A90V
H1395	ADC	*KEAP1*	KELCH1	c.1048G > A	p.G350S

SCLC, small cell lung cancer; LCC, large cell carcinoma; ADC, adenocarcinoma. BTB, Broad complex, tramtrack and bric-a-brac; IVR, the intervening linker domain; KELCH1-2-3, Kelch-repeat domains.

## Supplementary Materials

Supplementary materials can be found at https://www.mdpi.com/1422-0067/20/11/2697/s1. Supplemental Figure S1. Representative *KEAP1* promoter methylation pyrograms after DNA bisulfite conversion obtained with the first (1–7 CpGs, P1a region) and second reactions of primers (8–13 CpGs, P1b region) respectively. (A) Universal Methylated Human DNA; (B) Universal Unmethylated Human DNA. X axis shows the dispensation order; Percentages indicate the proportion of C (cytosine). Supplemental Table S1. (A) Pyrosequencing methylation levels at P1 *KEAP1* promoter region in normal MRC5 and BEAS-2B cell lines. (B) Pyrosequencing methylation level of each single CpG site at P1 *KEAP1* promoter region in normal MRC5 and BEAS-2B lung cell line.
