# Supplementary material for: Methylation Density Pattern of KEAP1 Gene in Lung Cancer Cell Lines Detected by Quantitative Methylation Specific PCR and Pyrosequencing
Source: Int J Mol Sci. 2019 May 31;20(11):2697. doi: 10.3390/ijms20112697 (PMC6600322; doi:10.3390/ijms20112697)
Supplement: Supplementary file 1 [file ijms-20-02697-s001.pdf]

Supplemental Materials

Supplemental Figure

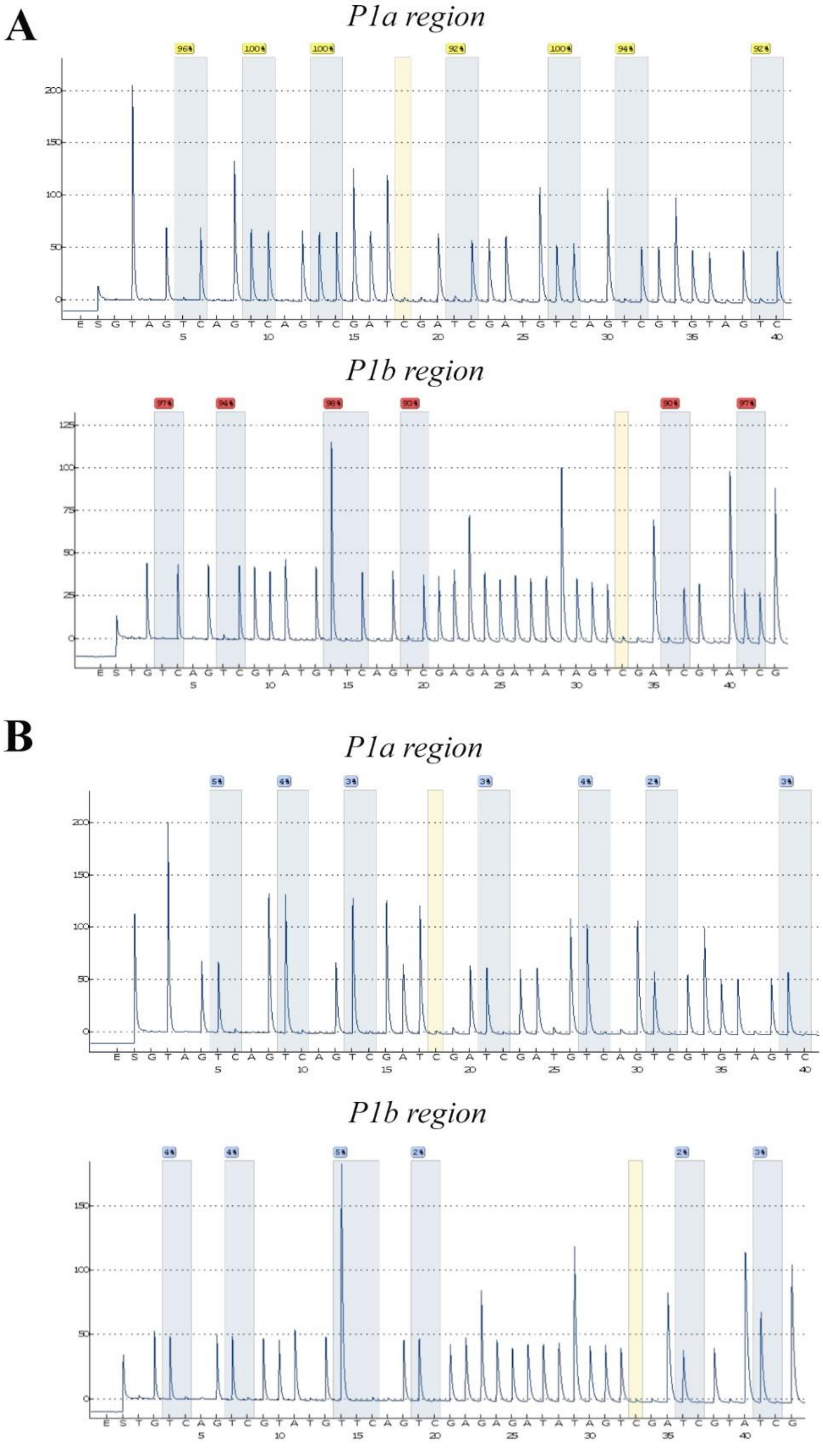

Supplemental Figure S1. Representative *KEAP1* promoter methylation pyrograms after DNA bisulfite conversion obtained with the first (1–7 CpGs, P1a region) and second reactions of primers (8–13 CpGs, P1b region) respectively. (**A**) Universal Methylated Human DNA; (**B**) Universal

Unmethylated Human DNA. X axis shows the dispensation order; Percentages indicate the proportion of C (cytosine).

Supplemental Table

Table S1 (**A**). Pyrosequencing methylation levels at P1 KEAP1 promoter region in normal cell lines. *methylation levels are reported as mean±SD of methylation levels of 13 CpGs mapped in the P1 promoter region. Each methylation value is expressed in %.

Cell Lines	Mean (%)*	2SD	Mean+2SD (%)*
MRC5	19.6	4.545327271	24.14532727
BEAS-2B	23.12	3.740053476	26.86005348

Table S1 (**B**). Pyrosequencing methylation level of each single CpG site at P1 *KEAP1* promoter region in normal lung cells.

Cell lines	CpG1	CpG2	CpG3	CpG4	CpG5	CpG6	CpG7	CpG8	CpG9	CpG 10	CpG 11	CpG 12	CpG 13
**MRC5**	37.0	34.6	39.2	17.0	22.0	23.8	17.8	17.6	16.4	11.2	8.8	4.6	4.8
**BEAS-2B**	56.4	32.8	40.4	13.6	51.8	49.0	20.6	8.8	9.6	7.4	4.6	2.6	3.2
**mean**	46.7	33.7	39.8	15.3	36.9	36.4	19.2	13.2	13.0	9.3	6.7	3.6	4.0
**2SD**	27.4	2.5	1.7	4.8	42.1	35.6	4.0	12.4	9.6	5.4	5.9	2.8	2.3
**mean+2SD**	74.1	36.2	41.5	20.1	79.0	72.0	23.2	25.6	22.6	14.7	12.6	6.4	6.3

*methylation values are as % for each 13 CpGs mapped in the P1 promoter region of KEAP1. Mean is referred to mean of values obtained for MRC5 and BEAS-2B by pyrosequencing.
